# Supplementary material for: The Systems Biology Research Tool: evolvable open-source software
Source: BMC Syst Biol. 2008 Jun 29;2:55. doi: 10.1186/1752-0509-2-55 (PMC2446383; doi:10.1186/1752-0509-2-55)
Supplement: Additional file 1 — SBRT Archive. An archive of the current version of the Systems Biology Research Tool. [file 1752-0509-2-55-S1.zip › sbrt-1.4.0/doc/developers_guide/api/sbrt/kernel/math/algebra/class-use/IndexPair.html]

Uses of Class sbrt.kernel.math.algebra.IndexPair


|  |  |  |
| --- | --- | --- |
| **Overview** \| **Package** \| **Class** \| **Use** \| **Tree** \| **Deprecated** \| **Index** \| **Help** | |  |
| PREV   NEXT | **FRAMES**    **NO FRAMES**     **All Classes** |  |


---


## **Uses of Class sbrt.kernel.math.algebra.IndexPair**

| Packages that use IndexPair | |
| --- | --- |
| **sbrt.kernel.fba.proc** | Provides classes and interfaces for executing Flux Balance Analysis processes. |
| **sbrt.kernel.math.algebra** | Provides classes and interfaces for creating objects relevant to algebra. |
| **sbrt.shell.text.math** | Provides classes and interfaces for defining formats for mathematical objects. |

Uses of IndexPair in sbrt.kernel.fba.proc

| Method parameters in sbrt.kernel.fba.proc with type arguments of type IndexPair | |
| --- | --- |
| `void` | `StoichVariationProcess.setMatrixSource(java.lang.Iterable<java.util.Map<IndexPair<java.lang.String,java.lang.String>,java.lang.Double>> matrixSource)`             Sets the stoiciometry variation input source. |

Uses of IndexPair in sbrt.kernel.math.algebra

| Methods in sbrt.kernel.math.algebra that return IndexPair | |
| --- | --- |
| `IndexPair<R,C>` | `MatrixElement.getIndices()`             Returns the pair of indices. |

| Method parameters in sbrt.kernel.math.algebra with type arguments of type IndexPair | |
| --- | --- |
| `void` | `AbstractSmdMatrix.setElements(java.util.Map<IndexPair<R,C>,java.lang.Double> elements)`             Sets the corresponding elements of this matrix to those provided. |

Constructors in sbrt.kernel.math.algebra with parameters of type IndexPair

`MatrixElement(IndexPair<R,C> indices,
U element)`
  
          Constructs a new matrix element.

Uses of IndexPair in sbrt.shell.text.math

Methods in sbrt.shell.text.math with type parameters of type IndexPair

`<S extends IndexPair<R,C>>
  
java.lang.String`

`IndexPairFormatV1.format(S indexPair)`
  
          Returns a formatted string representation of the
provided index pair.

| Methods in sbrt.shell.text.math that return IndexPair | |
| --- | --- |
| `IndexPair<R,C>` | `IndexPairFormatV1.parse(java.lang.String indexPair)`             Parses the provided string and returns its corresponding index pair. |

| Methods in sbrt.shell.text.math with parameters of type IndexPair | |
| --- | --- |
| `java.lang.String` | `IndexPairFormatV1.formatPair(IndexPair<? extends R,? extends C> indexPair)`             Returns a formatted string representation of the provided index pair. |

---


|  |  |  |
| --- | --- | --- |
| **Overview** \| **Package** \| **Class** \| **Use** \| **Tree** \| **Deprecated** \| **Index** \| **Help** | |  |
| PREV   NEXT | **FRAMES**    **NO FRAMES**     **All Classes** |  |


---
